# Leukocyte trafficking-associated vascular adhesion protein 1 is expressed and functionally active in atherosclerotic plaques

**DOI:** 10.1038/srep35089

**Published:** 2016-10-12

**Authors:** Johanna M. U. Silvola, Helena Virtanen, Riikka Siitonen, Sanna Hellberg, Heidi Liljenbäck, Olli Metsälä, Mia Ståhle, Tiina Saanijoki, Meeri Käkelä, Harri Hakovirta, Seppo Ylä-Herttuala, Pekka Saukko, Matti Jauhiainen, Tibor Z. Veres, Sirpa Jalkanen, Juhani Knuuti, Antti Saraste, Anne Roivainen

**Affiliations:** 1 Turku PET Centre, University of Turku, FI-20521 Turku, Finland; 2 Turku PET Centre, Turku University Hospital, FI-20521 Turku, Finland; 3 Turku Center for Disease Modeling, University of Turku, FI-20520 Turku, Finland; 4 Department of Vascular Surgery, Turku University Hospital and University of Turku, FI-20520 Turku, Finland; 5 A. I. Virtanen Institute for Molecular Sciences, University of Eastern Finland, FI-70211 Kuopio, Finland; 6 Department of Pathology and Forensic Medicine, University of Turku, FI-20520 Turku, Finland; 7 National Institute for Health and Welfare, Genomics and Biomarkers Unit, Biomedicum, FI-00251 Helsinki, Finland; 8 MediCity Research Laboratory and Department of Medical Microbiology and Immunology, University of Turku, FI-20520 Turku, Finland; 9 Heart Center, Turku University Hospital, FI-20520 Turku, Finland; 10 Institute of Clinical Medicine, University of Turku, FI-20520 Turku, Finland

## Abstract

Given the important role of inflammation and the potential association of the leukocyte trafficking-associated adhesion molecule vascular adhesion protein 1 (VAP-1) with atherosclerosis, this study examined whether functional VAP-1 is expressed in atherosclerotic lesions and, if so, whether it could be targeted by positron emission tomography (PET). First, immunohistochemistry revealed that VAP-1 localized to endothelial cells of intra-plaque neovessels in human carotid endarterectomy samples from patients with recent ischemic symptoms. In low-density lipoprotein receptor-deficient mice expressing only apolipoprotein B100 (LDLR^−/−^ApoB^100/100^), VAP-1 was expressed on endothelial cells lining inflamed atherosclerotic lesions; normal vessel walls in aortas of C57BL/6N control mice were VAP-1-negative. Second, we discovered that the focal uptake of VAP-1 targeting sialic acid-binding immunoglobulin-like lectin 9 based PET tracer [^68^Ga]DOTA-Siglec-9 in atherosclerotic plaques was associated with the density of activated macrophages (r = 0.58, *P* = 0.022). As a final point, we found that the inhibition of VAP-1 activity with small molecule LJP1586 decreased the density of macrophages in inflamed atherosclerotic plaques in mice. Our results suggest for the first time VAP-1 as a potential imaging target for inflamed atherosclerotic plaques, and corroborate VAP-1 inhibition as a therapeutic approach in the treatment of atherosclerosis.

Vascular adhesion protein-1 (VAP-1) is expressed on the endothelial cell surface and guides leukocytes to sites of inflammation. In addition to leukocyte binding, VAP-1 (currently known as a copper-containing amine oxidase AOC3, EC 1.4.3.6) has enzymatic activity. The latter is not only important for its adhesive function, but also modulates the inflammatory microenvironment by regulating chemokines, transcription factors, and other adhesion molecules, a process that can lead to cellular oxidative stress^1^,^2^,^3^,^4^.

Inflammation plays a role in the development of atherosclerosis and its associated complications^5^; however, the role of VAP-1 in atherosclerotic inflammation is unclear. VAP-1 gene expression is upregulated in atherosclerotic plaques in human carotid arteries^6^ and in the aorta of hypercholesterolemic rabbits^7^. Overexpression of human VAP-1 as a transgene on mouse endothelial cell leads to changes in the progression of atherosclerotic lesions^8^. Furthermore, levels of soluble VAP-1 are associated with risk factors for atherosclerosis^9^, carotid artery intimal thickening^10^, and clinical cardiovascular events^11^ in asymptomatic individuals.

Because [^18^F]FDG (2-[^18^F]fluoro-2-deoxy-*D*-glucose) accumulates in macrophages, positron emission tomography (PET) with this glucose analogue is used to image vascular inflammation^12^,^13^. However, uptake of [^18^F]FDG is not specific for inflammation; thus new imaging agents are called for. We previously developed and evaluated several imaging agents that target VAP-1 in various experimental animal models^14^,^15^,^16^,^17^,^18^. In addition, we used a phage-display method to identify a novel VAP-1-binding ligand, sialic acid-binding immunoglobulin-like lectin 9 (Siglec-9), which is expressed in activated monocytes and granulocytes. We then labeled a synthetic 1,4,7,10-tetraazacyclododecane-*N,N′,N″,N″′*-tetraacetic acid-conjugated Siglec-9 motif-containing peptide with ^68^Gallium ([^68^Ga]DOTA-Siglec-9) for PET imaging^15^.

Given the important role of inflammation in atherosclerosis, the present study aimed to investigate 1) whether functionally active VAP-1 is expressed in atherosclerotic plaques, and 2) whether the new VAP-1-targeting radioligand [^68^Ga]DOTA-Siglec-9 can detect inflamed atherosclerotic lesions.

## Results

### Characterization of atherosclerotic plaques in LDLR^−/−^ApoB^100/100^ mice

The basic characteristics of all animals are presented in Table 1. Atherosclerotic plaques in the aortas of LDLR^−/−^ApoB^100/100^ mice were mostly of the fibro-atheroma type, with a well-defined fibrous cap and macrophage infiltration (see Supplementary material Figure S1). The aortas of C57BL/6N control mice showed no evidence of atherosclerosis.

### Detection of VAP-1 in atherosclerotic plaques

**Figure 1:**
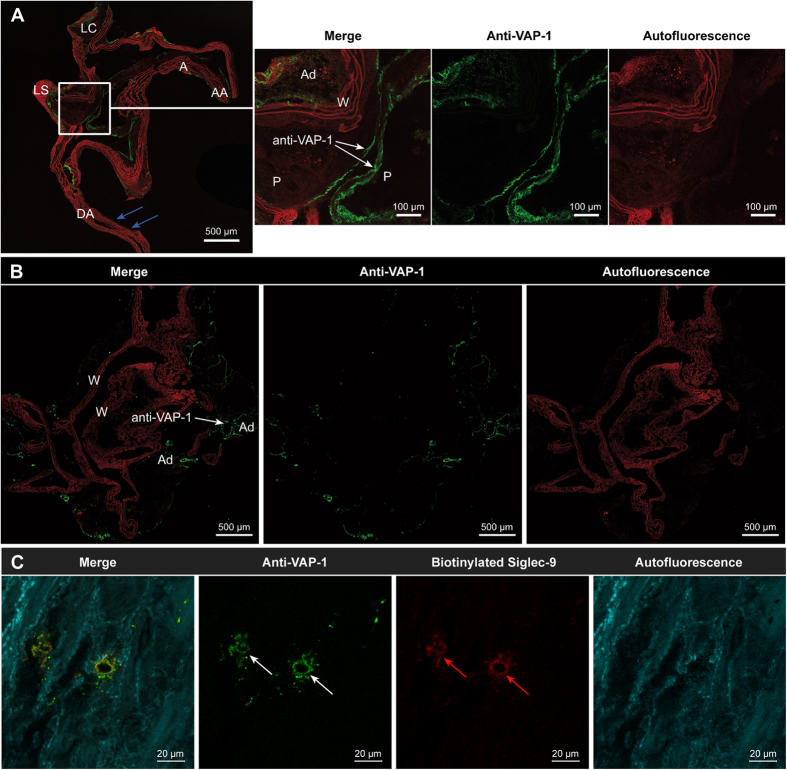
Vascular adhesion protein-1 (VAP-1) expression in atherosclerotic plaques. (**A**) VAP-1 expression was detected in luminal endothelial cells (green fluorescence; white arrows) lining the aortic plaques of low-density lipoprotein receptor-deficient mice expressing only apolipoprotein B100 (LDLR^−/−^ApoB^100/100^) after intravenous injection of an anti-VAP-1 antibody, followed by immunohistochemical detection with a fluorescent secondary antibody. Normal vessel walls in the same sections (blue arrows) were VAP-1-negative, indicating the specificity of VAP-1 for the endothelium in atherosclerotic plaques. The autofluorescence (red color) in elastic fibers was observed even in native adjacent sections that were not stained. The adipocytes around the vessel walls showed moderate VAP-1 staining. AA = ascending aorta, A = aortic arch, LC = left common carotid artery. LS = left subclavian artery, DA = descending aorta, P = plaque, W = wall, Ad = adipocyte. (**B**) The endothelium in healthy C57BL/6N control mice was mainly VAP-1-negative, whereas adipocytes were VAP-1-positive. (**C**) Sections of human carotid artery were double-stained with a biotinylated Siglec-9 peptide and an anti-VAP-1 antibody. The endothelial cell of small capillaries inside atherosclerotic plaques in the area of intima were highly VAP-1 positive (white arrows) and co-localized with the biotinylated sialic acid-binding immunoglobulin-like lectin 9 (Siglec-9) motif containing peptide (red arrows), as demonstrated by *in situ* immunohistochemistry methods.

The endothelial cells lining the walls of normal vessels or the intimal layers of plaques in atherosclerotic mice showed no binding of the i.v.-injected anti-VAP-1 antibody. By contrast, the VAP-1 antibody bound consistently to the surface of luminal endothelial cells lining the atherosclerotic plaques. Adipocytes around blood vessels in both atherosclerotic and control mice showed moderate levels of VAP-1-positive staining (Fig. 1A,B). Otherwise, the aortas of control mice were VAP-1-negative. There was no positive signal on the luminal surface of endothelial cells in the absence of the primary anti-VAP-1 antibody. The endothelial cells of intra-plaque neovessels in human carotid endarterectomy samples were VAP-1-positive, and VAP-1 co-localized with the biotinylated Siglec-9 peptide (Fig. 1C).

### Studies with VAP-1-targeting [^68^Ga]DOTA-Siglec-9

**Figure 2:**
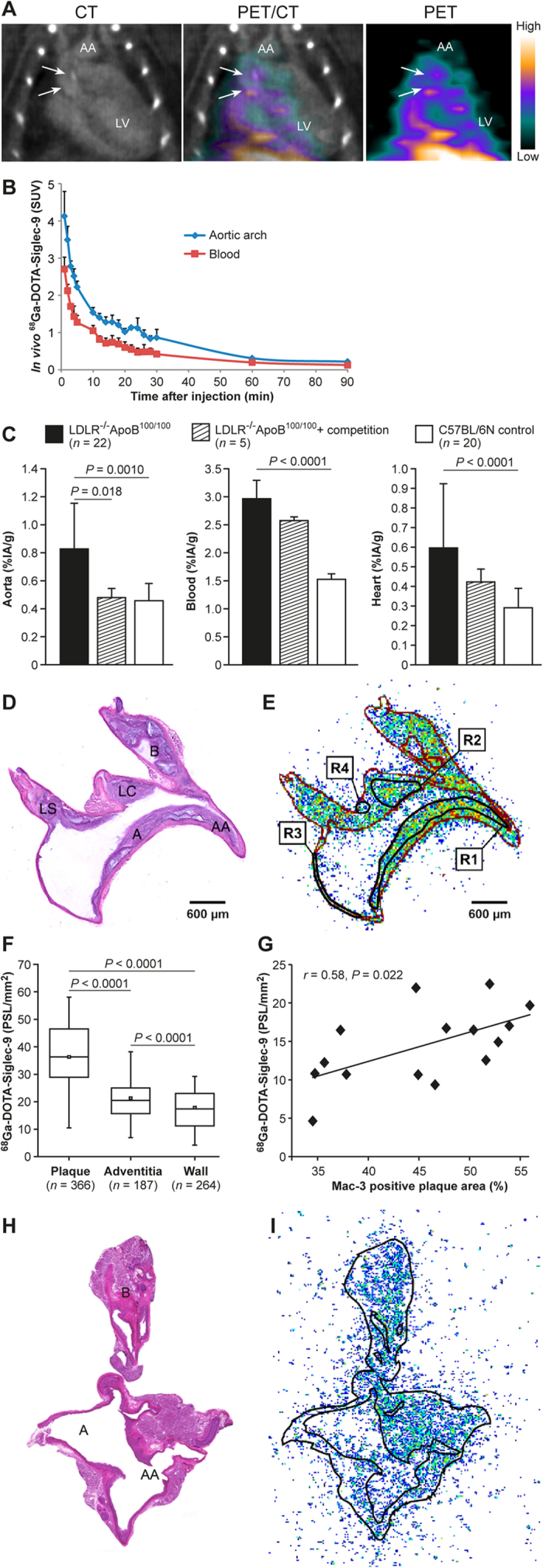
Accumulation of vascular adhesion protein-1 (VAP-1)-targeting sialic acid-binding immunoglobulin-like lectin 9-based radioligand ([^68^Ga]DOTA-Siglec-9) in atherosclerotic plaques. (**A**) Representative thoracic contrast-enhanced computed tomography (CT), positron emission tomography (PET)/CT and PET images of a LDLR^−/−^ApoB^100/100^ mouse at 10−20 min after tracer injection created using Inveon Research Workplace software (Siemens Medical Solutions, Knoxville, TN, USA) shows calcified atherosclerotic plaques (arrows) in the aortic arch (AA). The *in vivo* [^68^Ga]DOTA-Siglec-9 PET detects atherosclerotic plaques in the aortic root with target-to-background ratio (SUV_max, aortic arch_/SUV_mean, blood_) 2.0. LV = left ventricle cavity. SUV = standardized uptake value. (**B**) Time-activity curves in regions of interest of atherosclerotic mice. Lines represent mean and error bars the standard deviation (*n* = 3). (**C**) *Ex vivo* biodistribution results expressed as a percentage of injected radioactivity dose per gram of tissue (%IA/g). Values are mean and error bars standard deviation. *P* values, ANOVA with Tukey’s correction. (**D–G**) Autoradiography analysis of aorta cryosections. (**D**) Hematoxylin-eosin staining of a longitudinally sectioned LDLR^−/−^ApoB^100/100^ mouse aorta. (**E**) Superimposed autoradiograph and hematoxylin-eosin staining (red lines represent the borders of the hematoxylin-eosin image). R1 and R2 are regions of interest (ROI) in the plaque (excluding the media); R3 is the ROI in the normal vessel wall (no lesion formation); R4 is the ROI in the adventitia (mainly adipose tissue around the aorta). A = arch; AA = ascending aorta; B = brachiocephalic artery; LC = left common carotid artery; LS = left subclavian artery. (**F**) Quantitative autoradiography of LDLR^−/−^ApoB^100/100^ mice aorta (*n* = 22). *P* values, ANOVA with Tukey’s correction. (**G**) Pearson’s correlation analysis of tracer uptake and Mac-3-positive macrophage density in atherosclerotic plaques. PSL/mm^2^ = photostimulated fluorescence per square millimeter. (**H**) Hematoxylin-eosin staining of a longitudinally sectioned healthy C57BL/6N control mouse aorta. (**I**) Superimposed autoradiograph and hematoxylin-eosin staining (the black line represents the borders of the hematoxylin-eosin image).

Representative [^68^Ga]DOTA-Siglec-9 PET/CT images and time-activity curves are shown in Fig. 2A,B. Atherosclerotic lesions were detectable by PET at 10–20 min post-injection (Fig. 2A). The target-to-background ratio, i.e., SUV_max, aortic arch_/SUV_mean, blood_, was 1.7 ± 0.22 (*P* = 0.00029).

Uptake of i.v.-injected [^68^Ga]DOTA-Siglec-9 by the whole aorta was significantly higher in atherosclerotic mice (0.83 ± 0.33%IA/g) than in controls (0.46 ± 0.10%IA/g) (*P* = 0.0010). Competition by excess unlabeled peptide reduced the uptake of [^68^Ga]DOTA-Siglec-9 by the aortas of LDLR^−/−^ApoB^100/100^ mice by 42% (0.48 ± 0.066%IA/g; *P* = 0.018), reaching the same level as that in controls (Fig. 2C). Also, competition reduced the aorta-to-muscle ratio by 32% (from 1.7 ± 0.59 to 1.1 ± 0.38, *P* = 0.075). However, the radioactivity concentration in the blood fell by only 13% (from 3.0 ± 1.3 to 2.6 ± 0.9%IA/g, *P* = 0.51) (Fig. 2C).

Autoradiography revealed that uptake of [^68^Ga]DOTA-Siglec-9 by atherosclerotic plaques in LDLR^−/−^ApoB^100/100^ mice was higher than that by the normal vessel wall or adventitia (Table 2 and Fig. 2D–F). Competition by excess unlabeled peptide reduced the uptake in plaques by 48% (from 37 ± 16 to 17 ± 10 PSL/mm^2^; *P* = 0.026), whereas uptake in the normal vessel walls remained unchanged. Notably, uptake of [^68^Ga]DOTA-Siglec-9 in atherosclerotic plaques correlated with the density of Mac-3-positive macrophages (*r* = 0.58, *P* = 0.022; Fig. 2G). The areal percentage of the aortic plaques occupied by Mac-3-positive macrophages was high (52% ± 9.0%; range, 35–62%). For comparison, representative hematoxylin-eosin staining and [^68^Ga]DOTA-Siglec-9 autoradiography of healthy C57BL/6N control mouse aorta are presented in Fig. 2H,I.

### Functional activity of VAP-1 in atherosclerotic plaques

**Figure 3:**
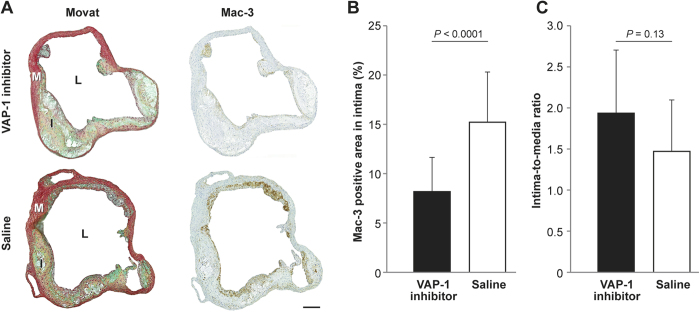
Effect of VAP-1 inhibition on atherosclerotic plaque inflammation in LDLR^−/−^ApoB^100/100^ mice. (**A**) Movat’s pentachrome (left column)- and Mac-3 (right column)-stained aortic ostium. I = intima, L = lumen, M = media. Scale bar, 200 μm. (**B**) Areal percentage of the aortic plaque occupied by Mac-3-positive macrophages and (**C**) intima-to-media ratio determined at the level of the aortic ostium after a 4 week treatment with the small molecular VAP-1 inhibitor, LJP1586 (*n* = 15), or saline (*n* = 10). Values are mean and error bars standard deviation. *P* values, non-paired *t* test.

Treatment with small molecular VAP-1 inhibitor LJP1586 for 4 weeks reduced the amount of Mac-3-positive macrophages in atherosclerotic plaques by 46% when compared with the saline group (inhibitor, 8.2% ± 3.5% *vs.* saline, 15% ± 5.1%; *P* < 0.0001; Fig. 3B). However, the inhibitor had no effect on plaque size, as determined by the intima-media ratio (inhibitor, 1.9 ± 0.8 *vs.* saline, 1.5 ± 0.6; *P* = 0.15; Fig. 3C), body weight, or plasma lipid levels (see Supplementary material, Table S1).

## Discussion

Here, we provide evidence that VAP-1 plays a role in inflammation associated with atherosclerosis, suggesting that VAP-1 is a potential target for imaging and for anti-inflammatory therapeutics used to treat vascular inflammation. First, we showed that VAP-1 is expressed on the luminal surface of endothelial cells in atherosclerotic plaques in LDLR^−/−^ApoB^100/100^ mice, and we confirmed a previous report showing expression of VAP-1 in human intra-plaque neovessels^19^. By contrast, endothelial cells lining non-atherosclerotic vessel walls were VAP-1-negative. Second, we discovered that focal uptake of VAP-1-targeting [^68^Ga]DOTA-Siglec-9 in atherosclerotic plaques is associated with the density of infiltrating macrophages. Finally, we found that inhibiting VAP-1 activity with small molecule LJP1586 reduced the macrophage density in atherosclerotic plaques in mice.

Several studies support the notion that adhesion molecules (e.g., P- and E-selectins, vascular cell adhesion molecule 1 (VCAM-1), and intercellular adhesion molecule 1 (ICAM-1)) play a role in the development of atherosclerosis; indeed, these molecules are expressed by endothelial cells lining atherosclerotic arteries in both humans and animals^20^,^21^,^22^. Recent studies also report VAP-1 expression in atherosclerotic lesions in humans and rabbits^6^,^7^. In contrast to many other leukocyte homing molecules, VAP-1 is practically absent from the surface of resting vascular endothelial cells; however, in an inflammatory environment, it is translocated rapidly from intracellular storage granules to the endothelial cell surface^1^,^2^,^17^, making it an attractive target for anti-inflammatory therapy and imaging.

The adhesive function of VAP-1 can be inhibited by certain enzymes^23^,^24^. Inhibiting VAP-1 reduces the migration of lymphocytes, granulocytes, and monocytes into inflamed tissues; it also reduces pathological angiogenesis in many animal models by abrogating monocyte/macrophage infiltration^25^,^26^. Here, we used a highly potent small molecular VAP-1 inhibitor, LJP1586 (mouse IC_50_ 4 nM)^27^, to demonstrate the functional importance of VAP-1 in atherosclerosis. The selectivity of LJP1586 for VAP-1 has been confirmed using a broad panel of receptors and enzymes, including monoamine oxidases A and B^27^. Marttila-Ichihara and co-workers showed that treatment of tumor-bearing mice with LJP1586 led to a significant reduction in the intratumoral accumulation of Gr-11CD11b1 myeloid cells and impaired tumor progression and neoangiogenesis^28^. Here, we found that treatment with LJP1586 led to a significant reduction in the density of macrophages in atherosclerotic plaques in mice, suggesting that VAP-1 is a potential target for therapeutics aimed at reducing inflammation associated with atherosclerosis. These results are in the line with previous mouse studies using a prototypic SSAO inhibitor, semicarbazide (also inhibiting lysyl oxidases and thus not being specific for VAP-1), as a treatment^29^,^30^. Mice with pre-existing, advanced atherosclerotic plaques were treated with VAP-1-targeted small molecule LJP1586 that probably explains that the short-term therapy at this stage had no effect on the size/amount of plaques. However, the finding that LJP1586 affected the extent of inflammation supports the finding of a previous study showing that inflammation is a more sensitive measure than the intima-to-media ratio^31^.

Another important finding is the high uptake of the VAP-1-targeting PET tracer, [^68^Ga]DOTA-Siglec-9, and its association with the density of Mac-3-positive macrophages in atherosclerotic plaques. The specificity of [^68^Ga]DOTA-Siglec-9 for VAP-1 was confirmed by a competitive binding assay in atherosclerotic mice, and further supported by the co-localization of Siglec-9 and an anti-VAP-1 antibody in human atherosclerotic plaques *in situ*. These observations are in the line with our previous studies, which verified that [^68^Ga]DOTA-Siglec-9 is specific for VAP-1 using three different approaches: 1) cell-binding assays using Chinese Hamster Ovary (CHO) cells stably transfected with human VAP-1 and CHO cells transfected with vector only; 2) *in vivo* biodistribution analysis in human VAP-1 transgenic mice and VAP-1 knockout mice; and 3) *in vivo* competition with excess of unlabeled Siglec-9 peptide in a mouse model of melanoma expressing VAP-1 in the vasculature. The latter model also provided an additional specificity control because VAP-1 knockout mice were available for the studies^15^.

*In vivo* [^68^Ga]DOTA-Siglec-9 PET/CT imaging showed a moderate signal in the aorta of atherosclerotic LDLR^−/−^ApoB^100/100^ mice, particularly in the aortic root (Fig. 2A). Humans express VAP-1 on the endothelial cells of intra-plaque neovessels; however, neovessels are rarely found in mice, which make *in vivo* imaging of atherosclerotic lesions in mice more challenging^32^. Thus, *in vivo* PET/CT imaging of human atherosclerosis-induced inflammation by targeting VAP-1 is probably achievable in humans.

Several non-invasive molecular imaging modalities have been used to detect different adhesion molecules involved in atherosclerotic inflammation^33^,^34^,^35^,^36^. To the best of our knowledge, only three papers report the imaging of atherosclerotic lesions using an adhesion molecule-targeting PET probe. Nahrendorf and co-workers report a [^18^F]4V PET probe, which allows *in vivo* detection of VCAM-1 in the mouse aortic root, with an aortic root-to-blood ratio of approximately 2 at 4 h post-injection; however, this tracer has not been evaluated in clinical studies^37^. Nakamura and co-workers report a [^64^Cu]DOTA-anti-P-selectin PET tracer, which allows *in vivo* detection of atherosclerotic mouse aortic root with a target-to-muscle ratio of 1.3 at 36 h post-i.v. injection^38^. However, the long physical half-life (12.7 h) of Cu^64^ results in high exposure to ionizing radiation, thereby limiting its use in humans. Very recently, Bala and co-workers reported a lesion-to-blood ratio of 3.3 at 3 h post-injection (*ex vivo* gamma counting) of a novel ^18^F-labeled anti-VCAM-1 nanobody^39^.

We previously used the same LDLR^−/−^ApoB^100/100^ mouse model and study protocols reported herein to perform a study with [^18^F]FDG^40^. Uptake of [^68^Ga]DOTA-Siglec-9 by aortic plaques, normal vessel wall, and adventitia was similar to that of [^18^F]FDG. Based on the autoradiography results, the plaque-to-normal vessel wall ratios were 2.1 ± 0.4 for [^68^Ga]DOTA-Siglec-9 *versus* 2.3 ± 0.5 for [^18^F]FDG (*P* = 0.44), respectively. According to the *ex vivo* biodistribution studies, uptake of [^68^Ga]DOTA-Siglec-9 in the heart was significantly lower, but blood radioactivity was significantly higher, than that of [^18^F]FDG. Based on the results presented herein, VAP-1 is a potential target for the imaging of atherosclerotic plaque inflammation. Although, *in vivo* detection of atherosclerotic plaques by [^68^Ga]DOTA-Siglec-9 PET/CT was moderate in mice, the results are in line with those presented in many other reports showing autoradiographic evidence of tracer accumulation, but limited *in vivo* imaging signals. This raises the question of whether mouse models are appropriate for preclinical evaluation of new tracers for clinical use. Furthermore, it may be that [^68^Ga]DOTA-Siglec-9 is not the optimal tracer for detecting VAP-1 *in vivo*, as a relatively high proportion of the injected radioactivity remains in the systemic circulation, at least in our LDLR^−/−^ApoB^100/100^ mice. The 25 min accumulation time-point was chosen based on our previous study^15^, and time-activity curves in Fig. 2B confirm this. Prolonging the accumulation time-point to 90 min did not improve the aorta-to-blood ratio in our atherosclerotic mouse model; therefore, it was not investigated further. In general, the lack of a sufficiently strong imaging signal can be explained by the level of target expression, the tracer contrast between the blood and vessel wall, partial volume effects, and the affinity of the radioligand for the protein. Additionally, the positron energy of the radionuclide used for PET affects both visualization and quantification. ^18^F emits low energy positrons (0.63 MeV), resulting in a very short positron range (0.27 mm in soft tissue), whereas ^68^Ga emits higher energy positrons (1.90 MeV) with a longer (1.05 mm) range^41^. Thus, further studies with ^18^F-labeled VAP-1 ligands(s) and larger atherosclerosis animal models are warranted.

In addition to endothelial cells of intra-plaque neovessels in human carotid arteries, VAP-1 is present on the endothelial cells lining inflamed atherosclerotic plaques, but not those in normal vessel walls, in mice. The uptake of the novel VAP-1-targeting imaging tracer [^68^Ga]DOTA-Siglec-9 by atherosclerotic plaques was associated with macrophage content. Finally, short-term treatment with LJP1586 reduced the macrophage density in atherosclerotic plaques in mice. Taken together, the results presented herein indicate that VAP-1 plays a role in inflammation associated with atherosclerosis and that it might be a potential target for imaging of inflamed atherosclerotic plaques and for anti-inflammatory therapeutics designed to treat vascular inflammation. As inhibition of VAP-1 reduces development of atherosclerotic lesions, one could envision that inhibition of inflammation may start the healing process and thus, reverse atherosclerosis.

## Methods

### Experimental animals and study design

The first phase examined VAP-1 expression in atherosclerotic plaques and the feasibility of using the VAP-1-targeting [^68^Ga]DOTA-Siglec-9 PET tracer to detect inflamed plaques. The tracer was administered to 6-month-old atherosclerotic, low-density lipoprotein receptor-deficient mice expressing only apolipoprotein B100 (LDLR^−/−^ApoB^100/100^, strain #003000; Jackson Laboratory, Bar Harbor, ME, USA) (*n* = 30) and then VAP-1 detected by immunohistochemistry and PET. Mice were fed a Western-type diet (42% of calories coming from fat and 0.2% from cholesterol, without sodium cholate; TD 88137, Harlan Teklad, Harlan Laboratories, Madison, WI, USA) for 4 months, starting at the age of 2 months and lasting up until the imaging experiments. Twenty 2-month-old C57BL/6N mice fed a regular chow diet served as healthy, non-atherosclerotic controls.

The second phase examined the role of VAP-1 in atherosclerotic plaques. Twenty-five LDLR^−/−^ApoB^100/100^ mice were treated with a VAP-1 inhibitor (LJP1586, Z-3-fluoro-2-(4-methoxybenzyl)allylamine hydrochloride; a kind gift from M. Linnik, La Jolla Pharmaceuticals, San Diego, CA, USA), or with saline. At the age of 2 months, mice were shifted to a high-fat diet for 2 months prior to the start of 4-week treatment, during which they were on normal chow. All mice were housed in an animal facility under standard conditions (12 h light/dark cycle) and allowed access to food and water *ad libitum*.

All animal experiments were approved by the National Animal Experiment Board in Finland and the Regional State Administrative Agency for Southern Finland, and carried out in accordance with the relevant European Union directives.

### Human carotid artery samples

Frozen human carotid endarterectomy samples (all containing atherosclerotic plaques) were obtained from patients (four female and one male; mean age 41 ± 9 years) with recent ischemic symptoms and double-stained with an anti-human VAP-1 antibody (10 μg/mL; JG2.10 gift from E. Butcher, Stanford University, CA, USA) and a biotinylated Siglec-9 peptide (20 μg/mL; NeoMPS, Strasbourg, France). The patient study was conducted in accordance with the Declaration of Helsinki, and the study protocol was approved by the Ethics Committee of Hospital District of Southwest Finland. All patients provided written informed consent.

### Characterization of plaques

Formalin-fixed, paraffin-embedded mouse hearts were transversely cut into 5 μm sections at the level of the coronary ostia and stained with an anti-Mac-3 antibody (Clone M3/84, dilution 1:5000, BD Biosciences, Franklin Lakes, NJ, USA) or modified Movat’s pentachrome stain, as previously described^40^. Stained sections were scanned with a Pannoramic 250 Flash digital slide scanner (3DHISTECH Ltd., Budapest, Hungary), and morphology was examined using Pannoramic Viewer 1.15 software (3DHISTECH Ltd.).

### Detection of VAP-1 in atherosclerotic plaques

VAP-1 expression in the aorta of high-fat fed LDLR^−/−^ApoB^100/100^ (*n* = 2) and C57BL/6N control mice (*n* = 2), and in carotid endarterectomy samples from patients with recent ischemic symptoms (*n* = 5), was examined by immunohistochemistry. To detect only luminal expression of VAP-1, mice were intravenously (i.v.) injected with a monoclonal rat anti-mouse VAP-1 antibody (7–88, 1 mg/kg diluted in saline)^2^ 10 min before sacrifice. Aorta samples were frozen and cut into 8 μm longitudinal sections, incubated for 30 min at room temperature in the dark with a secondary goat anti-rat antibody (working dilution, 5 μg/mL in phosphate-buffered saline (PBS) containing 5% normal mouse or human AB serum) conjugated to a fluorescent dye (Alexa Fluor 488; Invitrogen, Eugene, OR, USA), and rinsed twice in PBS for 5 min.

Co-localization of VAP-1 and the Siglec-9 motif peptide in atherosclerotic plaques was investigated in human carotid samples. Frozen endarterectomy sections (5 μm) were first incubated for 30 min with biotinylated Siglec-9 peptide (20 μg/mL; NeoMPS, Strasbourg, France) in Dulbecco’s PBS containing magnesium and calcium, and then detected with streptavidin-phycoerythrin. After three washes in PBS, the same sections were incubated with an anti-human VAP-1 antibody (JG2.10, 10 μg/mL) for 30 min, followed by detection with a fluorescein isothiocyanate-conjugated secondary immunoglobulin G antibody. Sections incubated in the absence of the Siglec-9 peptide served as a negative control. Tissue autofluorescence was distinguished from specific staining using the lambda scan mode and subsequent linear unmixing based on reference spectra. All fluorescent images were captured using a Zeiss LSM780 confocal microscope (Carl Zeiss MicroImaging GmbH, Jena, Germany).

### Studies using VAP-1-targeting [^68^Ga]DOTA-Siglec-9

#### Radiochemistry

A cyclic peptide harboring disulfide-bridged cysteines, CARLSLSWRGLTLCPSK, containing residues 283−297 from Siglec-9 and an 8-amino-3,6-diooxaoctanoyl linker (polyethylene glycol derivative) between 1,4,7,10-tetraazacyclododecane-*N*,*N*′,*N*″*N*″′-tetraacetic acid (DOTA) and the peptide (Peptide Specialty Laboratories GmbH, Heidelberg, Germany; C_104_H_174_N_30_O_32_S_2_; molecular weight, 2420.2 g/mol) was labeled with ^68^Ga, as described previously^15^. The radiochemical purity of [^68^Ga]DOTA-Siglec-9 was >96%, as analyzed by radiodetector-coupled high-performance liquid chromatography. The mean specific radioactivity was 55 MBq/nmol.

#### PET/CT imaging

A subset of LDLR^−/−^ApoB^100/100^ mice (*n* = 3) were subjected to *in vivo* imaging with a dedicated small-animal PET/CT scanner (Inveon Multimodality, Siemens Medical Solutions, Knoxville, TN, USA). Briefly, mice were anesthetized with isoflurane (induction 3.5%, maintenance 1.5%) and, after low dose CT for attenuation correction, i.v. injected with 5.3 ± 0.4 MBq of [^68^Ga]DOTA-Siglec-9 via a tail vein catheter. Dynamic PET data were acquired in list mode for 60 min (*n* = 2) or 90 min (*n* = 1), followed by contrast-enhanced CT angiography (eXIATM160XL, Binitio Biomedical Inc., Ottawa, ON, Canada), as described previously^22^. CT acquisition consisted of 161 projections with an exposure time of 1300 ms, an X-ray voltage of 80 kVp, and an anode current of 500 μA for a 220° rotation. The PET data were iteratively reconstructed using an ordered-subsets expectation maximization 3D algorithm (OSEM3D with four iterations and six subsets) to yield 5 × 60 s, 1 × 300 s, 10 × 120 s, and 2 × 1800 s time frames (matrix size, 128 × 128 × 159; pixel size, 0.776 × 0.776 × 0.796 mm). CT images were reconstructed using a Feldkamp-based algorithm (matrix size, 768 × 768 × 923; pixel size, 0.094 × 0.094 × 0.094 mm). Co-registration of PET and CT images was automatic and confirmed visually on the basis of anatomic landmarks. Quantitative PET image analysis was performed by defining regions of interest (ROI) within the aortic arch and the left ventricle of the heart (representing blood) using Inveon Research Workplace software (Siemens Medical Solutions, Malvern, PA, USA). Time frames 10–20 min post-injection were used for quantitative PET image analysis, and the results were expressed as standardized uptake values (SUVs) normalized for injected radioactivity dose and animal body weight, and as target-to-background ratios: SUV_max, aortic arch_/SUV_mean, blood_.

#### *Ex vivo* biodistribution

Mice (LDLR^−/−^ApoB^100/100^, *n* = 22; C57BL/6N, *n* = 20) under isoflurane anesthesia were i.v. injected with [^68^Ga]DOTA-Siglec-9 (19 ± 5 MBq). At 25 min post-injection, blood was drawn by cardiac puncture and the mice were sacrificed by cervical dislocation. The thoracic aorta was excised and rinsed in saline to remove blood. Aorta, blood, heart, and femoral muscle were weighed, and radioactivity was measured in a gamma counter (Triathler 3″, Hidex, Turku, Finland). Hearts were preserved in formalin for further characterization of plaques in the aortic root. The *ex vivo* biodistribution results were expressed as a percentage of the injected radioactivity dose per gram of tissue (%IA/g). The radioactivity remaining in the tail was compensated for.

For the competition study, isoflurane-anesthetized LDLR^−/−^ApoB^100/100^ mice were pre-injected with a 500-fold molar excess of unlabeled Siglec-9 peptide, which had an 8-amino-3,6-diooxaoctanoyl linker but no DOTA chelator (ChinaPeptides Co., Ltd. Shanghai, China), diluted in saline. Mice (*n* = 5) then received an injection of [^68^Ga]DOTA-Siglec-9 (22 ± 9 MBq) and were then subjected to subsequent *ex vivo* biodistribution and autoradiography analyses.

#### Autoradiography and support staining

The distribution of [^68^Ga]DOTA-Siglec-9 in the aorta was examined in more detail using autoradiography, as described previously^42^. Briefly, after gamma counting, excised aortas were frozen in isopentane, and sequential longitudinal cryosections (20 and 8 μm) were cut using a cryomicrotome at −15 °C. Sections were then thaw-mounted onto microscope slides. Next, the cryosections were apposed to an imaging plate (Fuji Imaging Plate BAS-TR2025; Fuji Photo Film Co., Ltd., Tokyo, Japan) and scanned after an exposure time of 2.5 h (Fuji Analyzer BAS-5000; Fuji, Tokyo, Japan; internal resolution, 25 μm).

The 20 μm sections were stained with hematoxylin and eosin and scanned with a Pannoramic 250 Flash slide scanner (3DHISTECH Ltd.), and morphology was examined using Pannoramic Viewer 1.15 software (3DHISTECH Ltd.). After carefully superimposing the autoradiographs and hematoxylin-eosin staining images, the concentration of ^68^Ga-radioactivity was measured in the following ROI: 1) plaques (excluding media); 2) normal vessel wall (no lesion formation); and 3) adventitia (mainly adipose tissue around the aorta). The results were expressed as count densities (photostimulated luminescence per square millimeter; PSL/mm^2^) using Tina 2.1 software (Raytest Isotopemessgeräte GmbH, Straubenhardt, Germany). The count density for background radiation was subtracted from the actual ROI data, and the results for each mouse were decay-corrected for injection time and exposure time and normalized for injected radioactivity dose. In total, 1142 ROIs (417 in plaques, 478 in normal vessel walls, and 347 in adventitia) were analyzed in 27 atherosclerotic and 20 control mice.

The 8 μm aorta cryosections were immunohistochemically stained with a rat anti-mouse Mac-3 antibody (Clone M3/84, dilution 1:1000, BD Biosciences, Franklin Lakes, NJ, USA) as described previously^43^ to compare the uptake of [^68^Ga]DOTA-Siglec-9 and the macrophage density in aortic plaques. Sections were scanned with a digital slide scanner (Pannoramic 250, 3DHISTECH Ltd.). The media was outlined and the Mac-3-positive area in each plaque was calculated (and expressed as %) using an automatic color deconvolution method and ImageJ (v. 1.46) software (Fiji, National Institutes of Health, Bethesda, MD, USA). Uptake of ^68^Ga-radioactivity in the same areas was evaluated using digital autoradiography.

### Assessment of functional activity of VAP-1 in atherosclerotic plaques

During the 4 week treatment period, LDLR^−/−^ApoB^100/100^ mice received thrice-weekly intraperitoneal (i.p.) injections of a small molecular VAP-1 inhibitor, LJP1586 (Z-3-fluoro-2-(4-methoxybenzyl)allylamine hydrochloride; 0.5 mg/mL diluted in PBS; a kind gift from M. Linnik, La Jolla Pharmaceuticals, San Diego, CA, USA) at a dose of 5 mg/kg. Mice injected with the physiological saline alone (200 μL) served as negative controls. After treatment, all mice were sacrificed and the hearts were collected, formalin-fixed, and paraffin-embedded. Tissues were then cut transversely into 5 μm sections at the level of the coronary ostia. The areas of intima and media were outlined in sections stained with Movat’s pentachrome, and the intima-to-media ratio for each mouse was determined with ImageJ as described previously^44^. Adjacent sections were stained with an anti-mouse Mac-3 antibody to detect activated macrophages in the intima as described above. Sections of aortic ostium were used to determine the area of plaque occupied by macrophages in each mouse because the ostium is easily located due to the presence of valves; it is therefore the most reliable place to compare plaque deposition in individual mice.

### Plasma assays

Total cholesterol (CHOD-PAP 1489232 kit; Roche Diagnostics GmbH, Mannheim, Germany), choline-containing phospholipids (990–54009; Wako Chemicals GmbH, Neuss, Germany and/or Diagnostic Systems, Holzheim, Germany), and triglycerides (GPO-PAP 1488872 kit; Roche Diagnostics GmbH) were measured using fully enzymatic methods.

### Statistical analyses

All results are expressed as the mean ± SD with two significant figures. Non-paired data were compared between two groups using a *t* test and between multiple groups using ANOVA with Tukey’s correction. A paired *t* test or Pearson’s correlation analysis was used to compare paired data between two groups. A *P* value <0.05 was considered statistically significant.

## Additional Information

**How to cite this article**: Silvola, J. M.U. *et al*. Leukocyte trafficking-associated vascular adhesion protein 1 is expressed and functionally active in atherosclerotic plaques. *Sci. Rep.* **6**, 35089; doi: 10.1038/srep35089 (2016).

## Supplementary Material

Supplementary Information

## Figures and Tables

**Table 1:** Characteristics of all investigated animals.

	LDLR−/−ApoB^100/100^	C57BL/6N control
Animal experiment I		
Animals, *n* (F/M)	30 (15/15)	20 (10/10)
Age, months	6	2
High-fat diet, months	4	ND
Weight, g (F/M)	25 ± 3.7/34 ± 5.6	21 ± 0.92/30 ± 3.3
*Ex vivo* and ARG, *n* (F/M)	22 (10/12)	20 (10/10)
PET/CT, *n* (F/M)	3 (0/3)	ND
Competition study*, *n* (F/M)	5 (5/0)	ND
Animal experiment II		
	VAP-1 inhibitor (LJP1586)	Saline
Animals†, *n* (F/M)	15 (8/7)	10 (7/3)
Age, months	5	5
High-fat diet, months	2	2
Weight, g (F/M)	25 ± 1.9/33 ± 1.7	23 ± 2.3/31 ± 2.1

F/M, female/male; *Ex vivo* and ARG, *ex vivo* gamma counting and autoradiography of [^68^Ga]DOTA-Siglec-9 uptake; ND, not done.

Body weight is expressed as the mean ± SD.

^*^Mice were pre-injected with excess unlabeled Siglec-9 peptide before injection of [^68^Ga]DOTA-Siglec-9, followed by *ex vivo* biodistribution and autoradiography studies at 25 min post-injection.

^†^LDLR^−/−^ApoB^100/100^ mice.

**Table 2:** Distribution of VAP-1-targeting [^68^Ga]DOTA-Siglec-9 in aortic cryosections, as determined by autoradiography.

	LDLR−/−ApoB^100/100^(*n* = 22)	LDLR−/−ApoB^100/100^ + competition (*n* = 5)	C57BL/6N controls(*n* = 20)
Plaque	37 ± 16	17 ± 10 *P* = 0.026*	ND
Wall	18 ± 9	10 ± 5 *P* = 0.086*	13 ± 7 *P* = 0.17†
Adventitia	21 ± 10	8 ± 4 *P* = 0.016*	14 ± 7 *P* = 0.017†
Plaque-to-wall	2.1 ± 0.43 *P* < 0.0001**	1.7 ± 0.13 *P* = NS**	ND
Plaque-to-adventitia	1.8 ± 0.35 *P* < 0.0001**	2.3 ± 0.75 *P* = NS**	ND
Wall-to-adventitia	0.83 ± 0.12 *P* < 0.0001**	1.3 ± 0.34 *P* = NS**	0.94 ± 0.16 *P* = 0.072**

Results are expressed as photostimulated luminescence per square millimeter or as ratios (mean ± SD). The competition assay was performed by pre-injecting unlabeled Siglec-9 peptide prior to injection of [^68^Ga]DOTA-Siglec-9.

ND, not determined.

^*^LDLR^−/−^ApoB^100/100 ^*vs.* LDLR^−/−^ApoB^100/100^ + competition, non-paired *t* test.

^†^LDLR^−/−^ApoB^100/100 ^*vs.* C57BL/6N controls, non-paired *t* test.

^**^P value, paired *t* test.
