# Supplementary material for: Leukocyte trafficking-associated vascular adhesion protein 1 is expressed and functionally active in atherosclerotic plaques
Source: Sci Rep. 2016 Oct 12;6:35089. doi: 10.1038/srep35089 (PMC5059718; doi:10.1038/srep35089)
Supplement: Supplementary Information [file srep35089-s1.pdf]

**Supplemental Material**

# Leukocyte trafficking-associated vascular adhesion protein 1 is expressed and functionally active in atherosclerotic plaques

Johanna M.U. Silvola[1,+], Helena Virtanen[1,2,+], Riikka Siitonen[1], Sanna Hellberg[1], Heidi Liljenbäck[1,3], Olli Metsälä[1], Mia Ståhle[1], Tiina Saanijoki[1], Meeri Käkelä[1], Harri Hakovirta[4], Seppo Ylä-Herttuala[5], Pekka Saukko[6], Matti Jauhiainen[7], Tibor Z. Veres[8], Sirpa Jalkanen[8], Juhani Knuuti[1,2], Antti Saraste[1,2,9,10], and Anne Roivainen[1,2,3,*]

[1]Turku PET Centre, University of Turku, FI-20521 Turku, Finland

[2]Turku PET Centre, Turku University Hospital, FI-20521 Turku, Finland

[3]Turku Center for Disease Modelling, University of Turku, FI-20520 Turku, Finland

[4]Department of Vascular Surgery, Turku University Hospital and University of Turku, FI-20520 Turku, Finland

[5]A. I. Virtanen Institute for Molecular Sciences, University of Eastern Finland, FI-70211 Kuopio, Finland

[6]Department of Pathology and Forensic Medicine, University of Turku, FI-20520 Turku, Finland

[7]National Institute for Health and Welfare, Genomics and Biomarkers Unit, Biomedicum, FI-00251 Helsinki, Finland

[8]MediCity Research Laboratory and Department of Medical Microbiology and Immunology, University of Turku, FI-20520 Turku, Finland

[9]Heart Center, Turku University Hospital, FI-20520 Turku, Finland

[10]Institute of Clinical Medicine, University of Turku, FI-20520 Turku, Finland

*corresponding author E-mail: anne.roivainen@utu.fi

+these authors contributed equally to this work

**Table S1. Plasma lipid levels of LDLR$^{-/-}$ApoB$^{100/100}$ mice treated with VAP-1 inhibitor LJP1586 or saline**

| | VAP-1 inhibitor LJP1586 (*n* = 15) | Saline (*n* = 10) | *P* value |
|---|---|---|---|
| Body weight, g | 28 ± 4.5 | 25 ± 4.3 | 0.065 |
| fP-Cholesterol | 8.8 ± 2.2 | 8.8 ± 1.3 | 0.99 |
| fP-HDL-cholesterol | 1.0 ± 0.25 | 1.0 ± 0.25 | 0.87 |
| fP-LDL-cholesterol | 7.1 ± 2.1 | 7.0 ± 1.4 | 0.83 |
| fP-Triglycerides | 1.5 ± 0.35 | 1.8 ± 1.0 | 0.24 |

Values are expressed as mean ± SD with two significant figures. LDLR$^{-/-}$ApoB$^{100/100}$, low-density lipoprotein receptor-deficient mouse expressing only apolipoprotein B100; VAP-1, vascular adhesion protein-1; fP, fasting plasma; HDL, high-density lipoprotein; LDL, low-density lipoprotein. Concentrations of lipids are expressed as mmol/L. *P* values, non-paired *t* test.

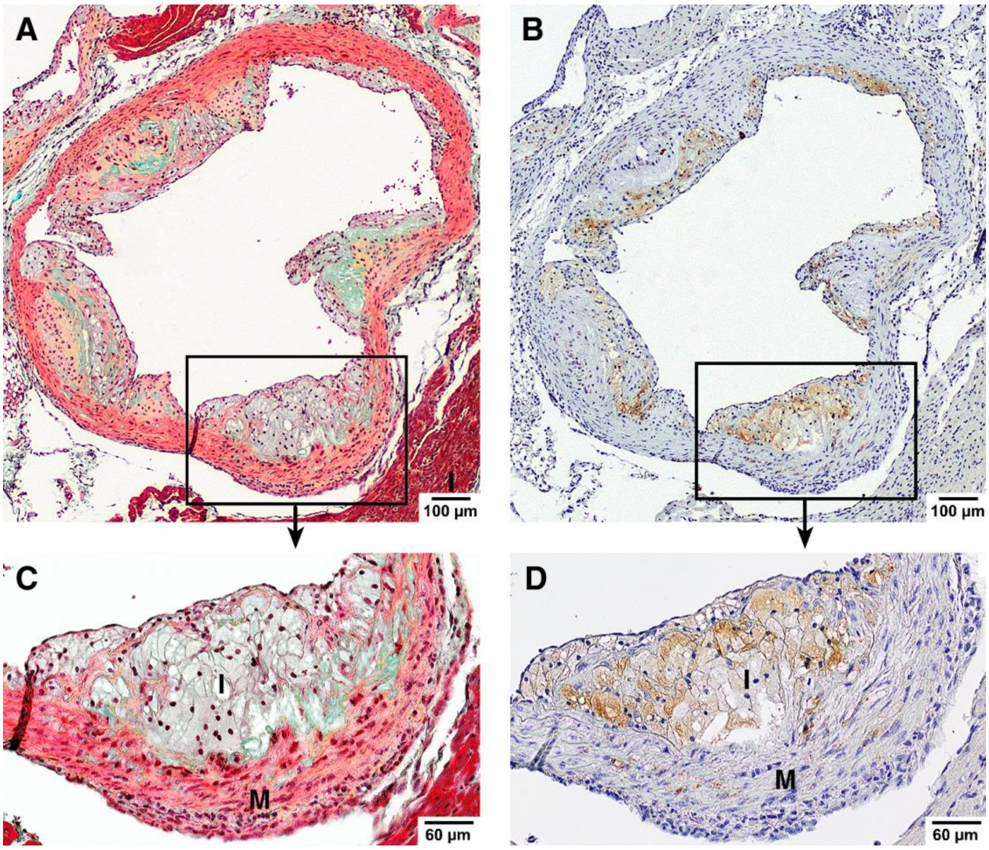


**Figure S1** Characterization of atherosclerotic plaques in LDLR$^{-/-}$ApoB$^{100/100}$ mice. The aortas of LDLR$^{-/-}$ApoB$^{100/100}$ mice contained atherosclerotic plaques, mostly of the fibroatheroma type with a well-defined fibrous cap. Representative formalin-fixed, paraffin-embedded aortic root sections stained with (A and C) Movat's pentachrome (black = nuclei; yellow = collagen, reticular fibers; blue = ground substance, mucin; bright red = fibrin; red = muscle) or with (B and D) anti-mouse Mac-3 immunohistochemistry in LDLR$^{-/-}$ApoB$^{100/100}$ mice. Mac-3 positive macrophages are represented by the brown color in images B and D. I = intima; M = media.
